# Polymorphisms in the *LPL* and *CETP* Genes and Haplotype in the *ESR1* Gene Are Associated with Metabolic Syndrome in Women from Southwestern Mexico

**DOI:** 10.3390/ijms160921539

**Published:** 2015-09-08

**Authors:** José Ángel Cahua-Pablo, Miguel Cruz, Abigail Méndez-Palacios, Diana Lizzete Antúnez-Ortiz, Amalia Vences-Velázquez, Luz del Carmen Alarcón-Romero, Esteban Juan Parra, Vianet Argelia Tello-Flores, Marco Antonio Leyva-Vázquez, Adán Valladares-Salgado, Claudia Paola Pérez-Macedonio, Eugenia Flores-Alfaro

**Affiliations:** 1 Laboratorio de Investigación en Epidemiología Clínica y Molecular, Unidad Académica de Ciencias Químico Biológicas, Universidad Autónoma de Guerrero, Chilpancingo, Guerrero 39089, Mexico; E-Mails: jose9@hotmail.com (J.A.C.-P.); abymendezp@hotmail.com (A.M.-P.); diana_antunez84@hotmail.com (D.L.A.-O.); avences_2003@yahoo.com.mx (A.V.-V.); vatellof@gmail.com (V.A.T.-F.); permac10@hotmail.com (C.P.P.-M.); 2 Unidad de Investigación Médica en Bioquímica, Hospital de Especialidades “Bernardo Sepúlveda”, Centro Médico Nacional Siglo XXI, Instituto Mexicano del Seguro Social, Distrito Federal 06725, Mexico; E-Mails: mcruzl@yahoo.com (M.C.); adanval@gmail.com (A.V.-S.); 3 Laboratorio de Biomedicina Molecular, Unidad Académica de Ciencias Químico Biológicas, Universidad Autónoma de Guerrero, Chilpancingo, Guerrero 39089, Mexico; E-Mails: luzdelcarmen14@gmail.com (L.C.A.-R.); leyvamarco13@gmail.com (M.A.L.-V.); 4 Department of Anthropology, University of Toronto at Mississauga, Mississauga, ON L5L 1C6, Canada; E-Mail: esteban.parra@utoronto.ca

**Keywords:** metabolic syndrome, *LPL* and *CETP* polymorphisms, *ESR1* haplotype

## Abstract

Metabolic syndrome (MetS) is a combination of metabolic disorders associated with an increased risk for cardiovascular disease (CVD). Studies in women reported associations between polymorphisms in *ESR1*, *LPL* and *CETP* genes and MetS. Our aim was to evaluate the association between variants in *ESR1*, *LPL* and *CETP* genes with MetS and its components. Four hundred and eighty women were analyzed, anthropometric features and biochemical profiles were evaluated, and genotyping was performed by real-time PCR. We found an association with elevated glucose levels (odds ratio (OR) = 2.9; *p* = 0.013) in carrying the AA genotype of rs1884051 in the *ESR1* gene compared with the GG genotype, and the CC genotype of rs328 in the *LPL* gene was associated with MetS compared to the CG or GG genotype (OR = 2.8; *p* = 0.04). Moreover, the GA genotype of rs708272 in the *CETP* gene is associated with MetS compared to the GG or AA genotype (OR = 1.8; *p* = 0.006). In addition the ACTCCG haplotype in the *ESR1* gene is associated with a decrease in the risk of MetS (OR = 0.02; *p* < 0.001). In conclusion, our results show the involvement of the variants of *ESR1*, *LPL* and *CETP* genes in metabolic events related to MetS or some of its features.

## 1. Introduction

Metabolic syndrome (MetS) is a combination of metabolic disorders and is associated with an increased risk for cardiovascular disease (CVD) and type 2 diabetes (T2D) in both genders [1]. The MetS traits, as defined by the guidelines the National Cholesterol Education Program Adult Treatment Panel III (NCEP-ATP III) most frequently used in the world include an increased waist circumference, blood pressure (BP) elevation, low high-density lipoprotein cholesterol (HDL-c), high triglycerides (TG) and hyperglycemia [2]. The NECP-ATP III provides evidence-based recommendations on the management of high blood cholesterol, metabolic syndrome and related disorders. For development of its recommendations, ATP III placed major emphasis on lifestyle changes as an essential modality in clinical management for persons at risk for CVD, as well as emphasis on large randomized controlled clinical trials [3,4]. Based on the ATP III guidelines, the diagnosis of MetS is performed when three or more of the five criteria, *i.e.*, abdominal obesity (waist circumference >88 cm for women or >102 cm for men), TG ≥ 150 mg/dL, reduced HDL-c < 50 mg/dL for women or <40 mg/dL for men, BP ≥ 130/85 mmHg and fasting glucose ≥110 mg/dL [2] are present. The incidence of MetS differs significantly between men and women, which has been attributed to differences in risk factors and hormone production [5].

Studies on associations in females have been reported for the estrogen receptor (*ESR1* or *ESR2*) with MetS or its components, particularly with obesity and dyslipidemia [6]; these associations might be explained by single nucleotide polymorphisms (SNPs) in the *ESR1* gene, as reported by some studies [7,8]. Moreover, SNPs in the lipoprotein lipase (*LPL*) and cholesteryl ester transfer protein (*CETP*) genes have been implicated in lipid abnormalities characteristic of MetS, where a decrease of the LPL is associated with hypertriglyceridemia and atherosclerosis, which, under normal conditions, hydrolyzes TG of chylomicrons and the very low density lipoproteins (VLDL), and CETP has the function of transferring cholesteryl esters from HDL to low density lipoprotein (LDL) as well as VLDL [9,10]. Given the close relationship between TG and HDL-c metabolism with various polymorphisms in the genes mentioned, the study of these SNPs in women from Southwestern Mexico may be of interest, due to the genetic diversity of populations. Therefore, our interest was to evaluate the association between SNPs and haplotypes in the *ESR1*, *LPL*, *CETP* genes with MetS or its components, in Mexican-Mestizo women. We found association between two polymorphisms in the *LPL* and *CETP* genes and a haplotype in the *ESR1* gene with MetS regardless of the admixture proportions.

## 2. Results

The average age of women participants was 46 years; 159 of them were diagnosed with MetS, with a prevalence of 33.1%. No significant differences were found in serum levels of atherogenic risk of LDL-c (≥160 mg/dL) among women with MetS compared to those who did not have the syndrome. Estimates of admixture proportions were obtained with the program ADMIXMAP v.3.8, using data from 104 ancestry informative markers (AIMs). The average Native American, European and African contributions were 69.2%, 27.1% and 3.7%, respectively, and were similar in women with or without MetS (Table 1).

**Table 1:** Somatometric, clinical and ancestry characteristics in women with and without MetS.

Characteristic	*n* = 480	MetS *n* = 159 (33.1%)	Without MetS *n* = 321 (66.9%)	*p*
Age (year)	46 (38–53)	51 (44–56)	43 (36–50)	<0.001 ^†^
BMI (kg/m^2^)	27.3 (24.8–30.3)	29.2 (27–33.4)	26.3 (24–29)	<0.001 ^†^
Abdominal obesity, *n* (%)	271 (56.5)	141 (88.7)	130 (40.5)	<0.001 ^‡^
% Body fat	36.6 (31.4–40.9)	39.5 (35.4–43.6)	35.4 (29.7–38.8)	<0.001 ^†^
% Water	44.4 (41.8–47.9)	42.2 (39.9–45.1)	45.3 (43–48.9)	<0.001 ^†^
BP systolic, (mm·Hg)	116 (107–127)	129 (117–137)	112 (105–120)	<0.001 ^†^
BP diastolic, (mm·Hg)	73.5 (67–81)	79 (71–86)	71 (66–78)	<0.001 ^†^
Glucose (mg/dL)	79.7 (71.5–89)	86 (77–107)	76.9 (70–83.4)	<0.001 ^†^
≥110, *n* (%)	54 (11.3)	43 (27)	11 (3.4)	<0.001 ^‡^
Total cholesterol (mg/dL)	172 (146.7–197)	184.3 (159.9–207)	165 (142–191.1)	<0.001 ^†^
≥200, *n* (%)	115 (24)	49 (30.8)	66 (20.6)	0.013 ^‡^
Triglycerides, (mg/dL)	130.5 (93.4–170.8)	170 (145–212.5)	110 (80.5–141)	<0.001 ^†^
≥150, *n* (%)	175 (36.5)	114 (71.7)	61 (19)	<0.001 ^‡^
HDL-c, (mg/dL)	39.9 (32.3–49.5)	37.6 (31.5–44.2)	40.4 (33.5–53.5)	<0.001 ^†^
<50, *n* (%)	366 (76.3)	147 (92.5)	219 (68.2)	<0.001 ^‡^
LDL-c, mg/dL	119 (90.5–157.2)	126.3 (95.3–168.3)	112.9 (88.8–155)	0.04 ^†^
≥160, *n* (%)	114 (23.9)	42 (26.8)	72 (22.5)	0.306 ^‡^
Exercise, *n* (%)	235 (49.1)	80 (50.6)	155 (48.3)	0.629 ^‡^
Years of schooling	17 (12–18)	17 (12–18)	17 (12–19)	0.155
Ancestry, %				
Native American	69.2	68.8	69.4	0.548 ^†^
European	27.1	27.7	26.7	0.275 ^†^
African	3.7	3.5	3.9	0.602 ^†^

Data are reported as medians (25th–75th percentile) or as noted in table. ^†^ Mann-Whitney test; ^‡^ Chi-square test; BMI: Body mass index; BP: Blood pressure; HDL-c: High density lipoprotein cholesterol; LDL-c: Low density lipoprotein cholesterol.

Table 2 shows the genotype distribution in women with and without MetS, as well as the comparison between the allelic frequencies obtained in the study with those reported in the database of SNPs of the National Center for Biotechnology Information (NCBI) (http://www.ncbi.nlm.nih.gov/projects/SNP/index.html).

**Table 2:** Genotype distribution and allele frequencies of the *ESR1*, *LPL* and *CETP* variants in the women with and without MetS, and allele frequencies reported by the database of SNP (dbSNP).

SNP	Total	Allele Frequencies	Allele Frequencies (dbSNP-NCBI)	MetS	Without MetS	*p* ^‡^
*ESR1*	*n* (%)			*n* (%)	*n* (%)	
rs1884051						
AA	95 (19.8)	A: 0.442	A = 0.509	29 (18.2)	66 (20.6)	0.251
AG	234 (48.7)	G: 0.558	G = 0.491	86 (54.1)	148 (46.1)	
GG	151 (31.5)			44 (27.7)	107 (33.3)	
rs3798577						
TT	142 (29.6)	T: 0.539	T = 0.536	44 (27.7)	98 (30.5)	0.740
CT	233 (48.5)	C: 0.461	C = 0.464	81 (50.9)	152 (47.4)	
CC	105 (21.9)			34 (21.4)	71 (22.1)	
rs2077647						
TT	146 (30.4)	T: 0.557	T = 0.533	49 (30.8)	97 (30.2)	0.991
CT	243 (50.6)	C: 0.443	C = 0.467	80 (50.3)	163 (50.8)	
CC	91 (19.0)			30 (18.9)	61 (19.0)	
rs1801132						
CC	190 (39.6)	C: 0.624	C = 0.718	64 (40.2)	126 (39.2)	0.975
CG	219 (45.6)	G: 0.376	G = 0.282	72 (45.3)	147 (45.8)	
GG	71 (14.8)			23 (14.5)	48 (15.0)	
rs2234693						
TT	270 (56.3)	T = 0.742 *	T = 0.554 *	93 (58.5)	177 (55.1)	0.254
CT	172 (35.8)	C = 0.258	C = 0.446	58 (36.5)	114 (35.5)	
CC	38 (7.9)			8 (5.0)	30 (9.4)	
rs9340799						
AA	295 (61.5)	A = 0.768	A = 0.719	102 (64.2)	193 (60.1)	0.309
AG	147 (30.6)	G = 0.232	G = 0.281	42 (26.4)	105 (32.7)	
GG	38 (7.9)			15 (9.4)	23 (7.2)	
*LPL*						
rs320						
TT	325 (67.7)	T = 0.821	T = 0.738	108 (67.9)	217 (67.6)	0.366
TG	138 (28.8)	G = 0.179	G = 0.262	48 (30.2)	90 (28.0)	
GG	17 (3.5)			3 (1.9)	14 (4.4)	
rs328						
CC	444 (92.5)	C = 0.961	C = 0.907	152 (95.6)	292 (91.0)	0.043
CG	35 (7.3)	G = 0.039	G = 0.093	6 (3.8)	29 (9.0)	
GG	1 (0.2)			1 (0.6)	0	
*CETP*						
rs708272						
GG	115 (24.0)	G = 0.493 *	G = 0.622 *	32 (20.1)	83 (25.9)	0.032
GA	243 (50.6)	A = 0.507	A = 0.378	94 (59.1)	149 (46.4)	
AA	122 (25.4)			33 (20.8)	89 (27.7)	

**^‡^** Chi-square test; * significant differences.

Significant differences were found in the allelic frequencies reported in the NCBI database in two SNPs (rs2234693 and rs708272) compared with those found in this investigation (*p* < 0.05). Genotype frequencies among women without MetS were consistent with the Hardy-Weinberg equilibrium (HWE) test. The highest frequencies of the minor allele (MAF) of polymorphisms in the *ESR1* gene corresponded to the rs1884051-G (55.8%), rs3798577-C (46.1%), rs2077647-C (44.3%), and rs1801132-G (37.6%), and the lowest frequencies to rs2234693-C (25.8%) and rs9340799-G (23.2%). The frequency of the minor allele of the rs708272-A in the *CETP* gene was 50.7%. The lowest frequency of the minor allele was found in the rs328-G (3.9%) in the *LPL* gene. No significant differences were found in the genotypic frequencies of the six SNPs in the gene *ESR1*, nor for rs320 SNP in the *LPL* gene (*p* > 0.05) in women who had MetS compared with those who did not have the syndrome. However, for the SNPs rs328 and rs708272 in the *LPL* and *CETP* genes respectively, significant differences were found (Table 2).

We found no association between SNPs in the *ESR1* gene with MetS, and only found significant association between women carrying the AA genotype of rs1884051 with elevated glucose levels (≥110 mg/dL) or previous diagnosis of T2D compared to the carriers of the GG genotype (OR = 2.9; 95% CI: 1.2–6.6; *p* = 0.013) (Table 3). Moreover, significant association was found between the CC genotype of rs328 in the *LPL* gene with MetS compared to women carrying the CG or GG genotype (OR = 2.8; 95% CI: 1.1–6.9; *p* = 0.04). In other words, women carrying the G allele (GC or GG) have lower risk of MetS, as shown in both models of inheritance: Co-dominant (OR = 0.3, 95% CI: 0.1–0.8, *p* = 0.018) and over-dominant (OR = 0.3, 95% CI: 0.1–0.8, *p* = 0.02) (Table 4). Furthermore, in a model of overdominant inheritance we found significant association of the GA genotype of rs708272 in the *CETP* gene with MetS compared to women carrying the GG or AA genotype (OR = 1.8; 95% CI: 1.2–2.7; *p* = 0.006) regardless of age, years of schooling and admixture proportion, where significant association was found with blood pressure ≥130/85 mmHg and glucose ≥110 mg/dL or T2D (Table 5).

**Figure 1:**
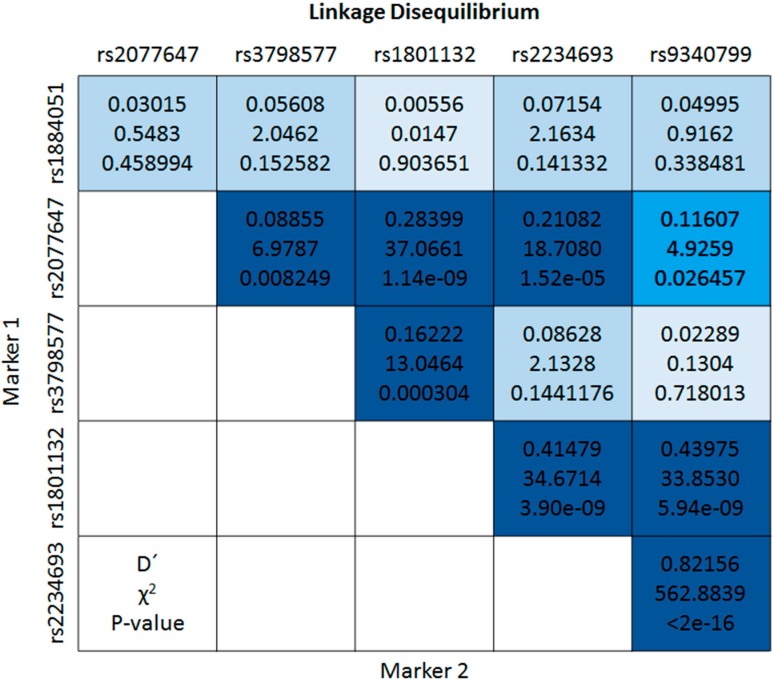
Map of linkage disequilibrium (LD) between the SNPs of the *ESR1* gene. The figure provided the statistics employed to determine the LD. The highest LD is indicated by navy blue (*p* < 0.0001 or *p* < 0.01), low disequilibrium show in medium blue (*p* < 0.05) and not significant in light blue (*p* > 0.05).

Fifty-two haplotypes were estimated from the six SNPs in the ESR1 gene, 27 (11.4%) of which had a lower frequency than 1% (rare), and the haplotype GCCCTA was the most frequent (0.097). A significant decrease in the risk of MetS was found with the ACTCCG haplotype (freq = 0.019) compared to the most frequent haplotype (OR = 0.02; 95% CI: 0.01–0.03; *p* < 0.001). The genetic association analyses (SNPs and haplotypes) were performed through logistic regression models adjusted for age, years of schooling and by the proportion of ancestry. Furthermore, we found strong linkage disequilibrium (LD) between five SNPs in the *ESR1* gene, greater LD between rs2234693 (Xbal) and rs9340799 (PvuII); the information is shown in Figure 1. Where the first value is the LD coefficient proposed by Lewontin (D′), the second value to the test of *X*^2^ and the third corresponds to the *p* values.

**Table 3:** Association between SNP rs1884051 in *ESR1* gene with MetS and their components.

Factor	Inheritance Model ^‡^														
	Co-Dominant							Dominant				Over-Dominant			
	GG	AG	OR (95% CI)	*p*	AA	OR (95% CI)	*p*	GG	AG + AA	OR (95% CI)	*p*	GG + AA	AG	OR (95% CI)	*p*
MetS, *n* (%)															
No	107 (33.3)	148 (46.1)	1.0		66 (20.6)	1.0		107 (33.3)	214 (66.7)	1.0		173 (53.9)	148 (46.1)	1.0	
Yes	44 (27.7)	86 (54.1)	1.3 (0.8–2.1)	0.237	29 (18.2)	0.9 (0.5–1.7)	0.768	44 (27.7)	115 (72.3)	1.2 (0.7–1.9)	0.430	73 (45.9)	86 (54.1)	1.4 (0.9–2.1)	0.125
AO > 88 cm, *n* (%)															
No	65 (31.1)	98 (46.9)	1.0		46 (22.0)	1.0		65 (31.1)	144 (68.9)	1.0		111 (53.1)	98 (46.9)	1.0	
Yes	86 (31.7)	136 (50.2)	1.0 (0.6–1.5)	0.890	49 (18.1)	0.7 (0.4–1.3)	0.768	86 (31.7)	185 (68.3)	0.9 (0.6–1.3)	0.599	135 (49.8)	136 (50.2)	1.1 (0.8–1.6)	0.653
BP ≥ 130/85 mm·Hg, *n* (%)															
No	119 (33.1)	172 (47.8)	1.0		69 (19.2)	1.0		119 (33.1)	241 (66.9)	1.0		188 (52.2)	172 (47.8)	1.0	
Yes	32 (26.7)	62 (51.6)	1.3 (0.8–2.1)	0.362	26 (21.7)	1.3 (0.7–2.4)	0.458	32 (26.7)	88 (73.3)	1.3 (0.8–2.0)	0.335	58 (48.3)	62 (51.6)	1.1 (0.7–1.8)	0.530
Glucose ≥110 mg/dL or T2D, *n* (%)															
No	140 (32.9)	209 (49.0)	1.0		77 (18.1)	1.0		140 (32.9)	286 (67.1)	1.0		217 (50.9)	209 (49.0)	1.0	
Yes	11 (20.4)	25 (46.3)	1.4 (0.7–3.1)	0.351	18 (33.3)	2.9 (1.2–6.6)	0.013	11 (20.4)	43 (79.6)	1.8 (0.9–3.7)	0.102	29 (53.7)	25 (46.3)	0.9 (0.5–1.5)	0.606
TG ≥ 150 mg/dL, *n* (%)															
No	97 (31.8)	140 (45.9)	1.0		68 (22.3)	1.0		97 (31.8)	208 (68.2)	1.0		165 (54.1)	140 (45.9)	1.0	
Yes	54 (30.9)	94 (53.7)	1.2 (0.8–1.8)	0.476	27 (15.4)	0.6 (0.4–1.1)	0.134	54 (30.9)	121 (69.1)	1.0 (0.7–1.5)	0.980	81 (46.3)	94 (53.7)	1.4 (0.9–2.0)	0.099
HDL-c < 50 mg/dL, *n* (%)															
No	34 (29.8)	49 (43.0)	1.0		31 (27.2)	1.0		34 (29.8)	80 (70.2)	1.0		65 (57.0)	49 (43.0)	1.0	
Yes	117 (32.0)	185 (50.5)	1.1 (0.7–1.8)	0.764	64 (17.5)	0.6 (0.3–1.0)	0.067	117 (32.0)	249 (68.0)	0.9 (0.6–1.4)	0.607	181 (49.5)	185 (50.5)	1.3 (0.9–2.1)	0.168

OR: Odds ratio; CI: Confidence interval; MetS: Metabolic syndrome; AO: Abdominal obesity; BP: Blood pressure; T2D: Type 2 diabetes; TG: Triglycerides; HDL-c: High density lipoprotein cholesterol. **^‡^** Model adjusted for age, years of schooling and by the admixture proportion. Considering a significance level (α) corrected = 0.01 (*p* < 0.01). Uncorrected *p* values are shown.

**Table 4:** Association between SNP rs328 in *LPL* gene with MetS and their components.

Factor	Inheritance Model ^‡^													
	Co-Dominant						Dominant				Over-Dominant			
	CC	CG	OR (95% CI)	*p*	GG	OR (95% CI)	CC	CG + GG	OR (95% CI)	*p*	CC + GG	CG	OR (95% CI)	*p*
MetS, *n* (%)														
No	292 (91.0)	29 (9.0)	1.0		0	1.0	292 (91.0)	29 (9.0)	1.0		292 (91.0)	29 (9.0)	1.0	
Yes	152 (95.6)	6 (3.8)	0.3 (0.1–0.8)	0.018	1 (0.6)	ND	152 (95.6)	7 (4.4)	0.4 (0.1–0.9) *	0.030	153 (96.2)	6 (3.8)	0.3 (0.1–0.8)	0.02
AO > 88 cm, *n* (%)														
No	192 (91.9)	16 (7.6)	1.0		1 (0.5)	1.0	192 (91.9)	17 (8.1)	1.0		193 (92.3)	16 (7.6)	1.0	
Yes	252 (93.0)	19 (7.0)	0.9 (0.4–1.7)	0.664	0	ND	252 (93.0)	19 (7.0)	1.3 (0.6–2.5)	0.529	252 (93.0)	19 (7.0)	0.9 (0.4–1.8)	0.670
BP ≥ 130/85 mm·Hg, *n* (%)														
No	329 (91.4)	31 (8.6)	1.0		0	1.0	329 (91.4)	31 (8.6)	1.0		329 (91.4)	31 (8.6)	1.0	
Yes	115 (95.8)	4 (3.4)	0.3 (0.1–1.0)	0.041	1 (0.8)	ND	115 (95.8)	5 (4.2)	2.5 (0.9–7.0)	0.080	116 (96.7)	4 (3.3)	0.3 (0.1–0.9)	0.040
Glucose ≥ 110 mg/dL or T2D, *n* (%)														
No	391 (91.8)	34 (8.0)	1.0		1 (0.2)	1.0	391 (91.8)	35 (8.2)	1.0		392 (92.0)	34 (8.0)	1.0	
Yes	53 (98.1)	1 (1.9)	0.2 (0.1–1.5)	0.115	0	ND	53 (98.1)	1 (1.9)	5.5 (0.7–42.4)	0.102	53 (98.1)	1 (1.9)	0.2 (0.1–1.5)	0.116
TG ≥ 150 mg/dL, *n* (%)														
No	280 (91.8)	25 (8.2)	1.0		0	1.0	280 (91.8)	25 (8.2)	1.0		280 (91.8)	25 (8.2)	1.0	
Yes	164 (93.7)	10 (5.7)	0.7 (0.3–1.4)	0.292	1 (0.6)	ND	164 (93.7)	11 (6.3)	1.4 (0.7–3.1)	0.364	165 (94.3)	10 (5.7)	0.6 (0.3–1.4)	0.289
HDL-c < 50 mg/dL, *n* (%)														
No	102 (89.5)	12 (10.5)	1.0		0	1.0	102 (89.5)	12 (10.5)	1.0		102 (89.5)	12 (10.5)	1.0	
Yes	342 (93.4)	23 (6.3)	0.5 (0.2–1.1)	0.090	1 (0.3)	ND	342 (93.4)	24 (6.6)	1.8 (0.9–3.8)	0.112	343 (93.7)	23 (6.3)	0.5 (0.2–1.1)	0.089

OR: Odds ratio; CI: Confidence interval; MetS: Metabolic syndrome; ND: Not determined; AO: Abdominal obesity; BP: Blood pressure; T2D: Type 2 diabetes; TG: Triglycerides; HDL-c: High density lipoprotein cholesterol. **^‡^** Model adjusted for age, years of schooling and by the admixture proportion. * The association between CC genotype with MetS was OR = 2.8 (95% CI: 1.1–6.9; *p* = 0.030) compared with CG or GG genotype. Considering a significance level (α) corrected = 0.01 (*p* < 0.01). Uncorrected *p* values are shown.

**Table 5:** Association between SNP rs708272 in *CETP* gene with MetS and their components.

Factor	Inheritance Model ^‡^														
	Co-Dominant							Dominant				Over-Dominant			
	AA	GA	OR (95% CI)	*p*	GG	OR (95% CI)	*p*	AA	GA + GG	OR (95% CI)	*p*	AA + GG	GA	OR (95% CI)	*p*
MetS, *n* (%)															
No	89 (27.7)	149 (46.4)	1.0		83 (25.9)	1.0		89 (27.7)	232 (72.3)	1.0		172 (53.6)	149 (46.4)	1.0	
Yes	33 (20.8)	94 (59.1)	1.7 (1.0–2.8)	0.042	32 (20.1)	0.9 (0.9–1.7)	0.749	33 (20.8)	126 (79.2)	1.4 (0.9–2.3)	0.170	65 (40.9)	94 (59.1)	1.8 (1.2–2.7)	0.006
AO > 88 cm, *n* (%)															
No	53 (25.3)	99 (47.4)	1.0		57 (27.3)	1.0		53 (25.3)	156 (74.6)	1.0		110 (52.6)	99 (47.4)	1.0	
Yes	69 (25.5)	144 (53.1)	1.1 (0.7–1.7)	0.794	58 (21.4)	0.7 (0.4–1.2)	0.794	69 (25.5)	202 (74.5)	0.9 (0.6–1.4)	0.769	127 (46.9)	144 (53.1)	1.2 (0.9–1.8)	0.250
BP ≥ 130/85 mm·Hg, *n* (%)															
No	103 (28.6)	168 (46.7)	1.0		89 (24.7)	1.0		103 (28.6)	257 (71.4)	1.0		192 (53.3)	168 (46.7)	1.0	
Yes	19 (15.8)	75 (62.5)	2.5 (1.4–4.4)	0.002	26 (21.7)	1.5 (0.7–2.9)	0.279	19 (15.8)	101 (84.2	2.1 (1.2–3.7)	0.009	45 (37.5)	75 (62.5)	2.0 (1.3–3.2)	0.002
Glucose ≥ 110 mg/dL or T2D, *n* (%)															
No	117 (27.5)	211 (49.5)	1.0		98 (23.0)	1.0		117 (27.5)	309 (72.5)	1.0		215 (50.5)	211 (49.5)	1.0	
Yes	5 (9.3)	32 (59.2)	3.5 (1.3–9.3)	0.013	17 (31.5)	3.7 (1.3–10.7)	0.016	5 (9.3)	49 (90.7)	3.6 (1.4–9.3)	0.010	22 (40.7)	32 (59.2)	1.5 (0.8–2.8)	0.161
TG ≥ 150 mg/dL, *n* (%)															
No	81 (26.6)	148 (48.5)	1.0		76 (24.9)	1.0		81 (26.6)	224 (73.4)	1.0		157 (51.5)	148 (48.5)	1.0	
Yes	41 (23.4)	95 (54.3)	1.2 (0.7–2.0)	0.375	39 (22.3)	0.9 (0.5–1.6)	0.684	41 (23.4)	134 (76.6)	1.1 (0.7–1.8)	0.630	80 (45.7)	95 (54.3)	1.3 (0.9–1.9)	0.171
HDL-c < 50 mg/dL, *n* (%)															
No	26 (22.8)	64 (56.1)	1.0		24 (21.1)	1.0		26 (22.8)	88 (77.2)	1.0		50 (43.9)	64 (56.1)	1.0	
Yes	96 (26.2)	179 (48.9)	0.8 (0.4–1.3)	0.285	91 (24.9)	1.0 (0.6–1.9)	0.913	96 (26.2)	270 (73.8)	0.8 (0.5–1.4)	0.460	187 (51.1)	179 (48.9)	0.7 (0.5–1.1)	0.164

OR: Odds ratio; CI: Confidence interval; MetS: Metabolic syndrome; AO: Abdominal obesity; BP: Blood pressure; T2D: Type 2 diabetes; TG: Triglycerides; HDL-c: High density lipoprotein cholesterol. ^‡^ Model adjusted for age, years of schooling and by the admixture proportion. Considering a significance level (α) corrected = 0.01 (*p* < 0.01). Uncorrected *p* values are shown.

## 3. Discussion

MetS is defined by a constellation of an interconnected physiological, biochemical, clinical, and metabolic factors that directly increase the risk of atherosclerotic cardiovascular disease, T2D, and all-cause mortality [11]. The prevalence of MetS in the world is high and significantly elevated in Latin America. Low prevalence has been reported in individuals with Dominican ancestry (14%) and higher in the Brazilian population [12]. In the study by Hess *et al.* in a population of Hispanic/Latino of diverse origin, they found a prevalence of 36% among women and 34% in men, among women the metabolic syndrome prevalence ranged from 27% in South Americans to 41% in Puerto Ricans [13]. In our study we found a prevalence of MetS of 33.1%, noting that it was lower than that reported in the country (42.2%) [14]. A number of potential candidate genes have been suggested according to their biological relevance and many of these have been strongly associated with MetS in different populations. In this study the relationship between SNPs in the *ESR1*, *LPL* and *CETP* genes with MetS and its components was analyzed, in women from Southwestern Mexico.

The genetic association has been described for SNPs in the *ESR1* gene with various pathological conditions, including CVD, T2D, hypertension, and lipoprotein metabolism [15,16]. Estrogen is a major effector for the regulation of energy balance, body weight, fat distribution, and appetite in mice. ESR1 has been shown to be involved in the maintenance of glucose metabolism in several tissues including liver, skeletal muscle, adipose tissue and pancreatic β cells. The critical role of ESR1 in maintaining glucose homeostasis has been validated in *Ob*/*Ob* mice where treatment with the ESR1-selective ligand propyl pyrazole triol improved glucose tolerance and insulin sensitivity. Moreover, estrogen modulates leptin (LEP) synthesis and secretion via ESR1-dependent transcriptional mechanisms. Form b (LEPRb) is the critical variant for regulating energy balance, it has been reported that the LEPRb is co-localized with ESR1 in the hypothalamus and regulates the expression of LEPRb mRNA via an estrogen response element in the LEPR gene, suggesting interactions between these pathways for the regulation of energy homeostasis. The exact mechanisms behind the estrogenic effects on leptin signaling and LEPRb levels are currently not well understood. Alterations in ESR1 affect leptin signaling downstream of LEPRb transcription/translocation. Changes in the structure and functionality of ESR1 may result from SNPs in the *ESR1* gene [15].

It has been shown previously that alternative splicing may result in variants with deletions of exons encoding regions of the hormone-binding domain with truncated forms of ESR1 discovered in many tissues including vascular endothelium and showing altered ligand-activation properties. We found strong linkage disequilibrium between five SNPs in the *ESR1* gene [17]. These SNPs are located in regions that affect the transcription of *ESR1*. The SNP rs2077647 is a silent polymorphism located in exon 1 (S10S), this location corresponds to the A/B structural domain, contains a co-regulator domain that binds coactivators or co-repressors to the ESR1, with an important role for the modulation of *ESR1* transcription [18]. The rs2234693 (Xbal) and rs9340799 (PvuII) located in intron 1 are in a strong LD; the possible functional mechanism that is attributed to these polymorphisms includes a change in the *ESR1* gene expression by an alteration in the binding of transcription factors and influence alternative splicing *ESR1* gene [6]. The rs1801132 is a silent polymorphism in codon 325 (325Pro) of exon 4, and represents a target for the exonic splicing enhancers sc35 and sf2 (arginine serine-rich splicing factors) that interact with small nucleolar RNA and are required for the first step in the splicing reaction and spliceosome assembly [18,19]. The rs3798577 polymorphism is located in the 3′-UTR of ESR1, and although its functionality is not yet known, taking into account that the 3′-UTR region is associated with the preferred target for microRNAs and splicing factors, it appears to modulate *ESR1* expression [20].

In the present study, we found no association of SNPs in the *ESR1* gene with MetS; these results coincide with those reported by Goulart *et al.,* where they found no relation of SNPs in *ESR1* and *ESR2* genes with MetS in Caucasian postmenopausal women (*n* = 532) [21]. However, in the study conducted in young women (*n* = 354) by Rebelo *et al.*, they found no association between rs2234693 and rs9340799 SNPs with metabolic variables (total cholesterol, HDL-c, LDL-c, and triglycerides) [22]. Moreover Gallagher *et al.* reported the association of rs9340799 polymorphism in the *ESR1* gene with MetS, and the C allele of rs2234693 with reduced insulin sensitivity in African American families (*n* = 548) [6]. In our study, only the AA genotype of SNP rs1884051 was found to be associated with elevated glucose levels, indicating that this variant may contribute to insulin resistance.

In the haplotype analysis for the six SNPs of the *ESR1* gene, we found a significant decrease in the risk of MetS attributed to haplotype ACTCCG (*p* < 0.001); the study of Gallagher *et al.* reported no significant association between haplotypes with MetS [6]. As mentioned, Gallagher *et al.* derived from other studies in animal models of the *ESR1* gene, supports pleiotropic effects on phenotypes related to diabetes and CVD risk because male and female *ESR1* knockout mice exhibit insulin resistance, impaired glucose tolerance, and obesity. A human male with an *ESR1*-null mutation had insulin resistance, impaired glucose tolerance and obesity [6].

The LPL and CETP enzymes participate in lipid metabolism; LPL catalyzes the hydrolysis of TG of VLDLs and chylomicrons and enhances the HDL-c level by processing HDL to its mature form, while CETP facilitates the transfer of cholesteryl esters from HDL to apolipoprotein (Apo) B-containing lipoproteins (VLDL, LDL), and exchanges them for cholesteryl esters from HDL. It has been reported that a deficiency of LPL and CETP enzymes are related to the development of atherosclerosis; this deficiency could be related to mutations or variants in their genes, or by altering molecules involved in its metabolism [10,23].

Several studies have found an association of SNP rs328 (S447X) in the *LPL* gene with lipid levels. Ariza *et al.* (*n* = 1825) found a significant decrease in serum TG levels in subjects carrying the GG (XX) genotype of rs328 [24]; similar results were reported by Webster *et al.* (*n* = 2864) where they indicated that the 447X (G) allele at the rs328 was associated with reduced TG levels (*p* = 0.0004) and raised HDL-c levels (*p* = 0.0004). The authors mention that their results are limited to people with European ancestry, and although many common variants have been identified that influence metabolic traits and diabetes risk, because they only studied four variants in three genes involved in T2D, additional studies are required [25]. On the other hand, contrary to the studies described above, Emamian *et al.* found that CG + GG genotypes were significantly associated with increased concentrations of TG (*p* = 0.044), mentioning that one of the main limitations of the study was the modest sample size (*n* = 271) [26]. In our study, we found significant association (*p* = 0.04) between women carrying the CC genotype rs328 with MetS compared with the carriers of the GG or CG genotypes, finding association of this genotype with high blood pressure, high levels of glucose and triglycerides, to have no statistical significance. We found low frequency of the GG genotype (3.5%), which may be a limiting factor, so further studies are needed with a larger sample size to confirm this association.

A number of SNPs have been described in the *CETP* gene. Most of these SNPs are associated with reduced plasma CETP and HDL-c concentrations. Interestingly, environmental factors have been shown to contribute to the association strength between these SNPs in the *CETP* gene and HDL-c concentration. It has been reported that the SNP rs708272 (TaqIB) in the *CETP* gene influences CETP, HDL-c concentration, and LDL-size. Carriers of the A (B2) allele of this polymorphism have been associated with significantly decreased CETP concentrations, raised HDL-c levels, and increased LDL-particle diameter [27]. Lopez-Rios *et al.*, found in a population of Spain (*n* = 457), that the GG (B1B1) genotype carriers showed significantly lower HDLc concentrations than the B2-allele (A) carriers (*p* < 0.001), as well as higher glucose levels after the oral glucose tolerance test [28]. Similar results were found by Elsammak *et al.* (*n* = 65) who reported similar genotypic frequencies to our study, and that serum levels of HDL-c was significantly higher in subjects with the AA (B2B2) genotype than those with B1B1 genotype. Additionally, they found a significant difference in the distribution of the different genotypes of the Taq1B polymorphism between controls and patients with metabolic syndrome (*p* = 0.03) [29]. In this study we found, in a model of overdominant inheritance, an increased risk of MetS in women carrying the GA genotype (OR = 1.8; 95% CI: 1.2–2.8; *p* = 0.006) compared with carriers of AA or GG genotypes, even after adjusting for confounding factors such as age, years of schooling and by the admixture proportion (Native American, European and African). This may mean that being homozygous for both alleles confers protection for the development of MetS, as indicated by our results, and that having a copy for both alleles (heterozygous) alters this protection. Experimental studies are needed to confirm or refute this hypothesis.

One of the limitations in our study is the small sample size, decreasing the statistical power to find the association between MetS and its components with the studied polymorphisms in the *ESR1* gene, as has been reported in other studies. The effects of genetic variants on phenotypic traits or diseases are generally small. However, it is possible that a set of genetic polymorphisms, together with environmental factors, contribute to modulation of the increased risk of development of diseases such as MetS. Moreover, it is possible that the contradictory results between studies are due to genetic diversity among populations.

## 4. Experimental Section

### 4.1. Subjects

A cross-sectional study was conducted in 480 female university workers, older than 30 years, residents and native of Guerrero, Mexico, a state located in the southwestern part of the country, whose parents and grandparents were also born in Guerrero. Pregnant women, with myocardial infarction, angina pectoris, cancer or hormone replacement therapy, were excluded. The project was approved by the Ethics Committee of the Autonomous University of Guerrero. All women agreed to participate in the study by means of written informed consent. Measurements of weight, height, waist circumference (WC) and blood pressure (BP) were performed on each of the women participating in the study. Weight was measured with the body composition monitor BC554 (Tanita Corporation, Tokio, Japan), height with mobile stadiometer m-217 (Seca, Hamburg, Germany), WC with anthropometric tape m-201 (Seca, Hamburg, Germany), and BP with BP monitor 3 AC1-PC (Microlife USA, Inc., Clearwater, FL, USA). Venous blood samples were obtained after fasting for 12 h for biochemical measurements and DNA extraction.

### 4.2. Diagnosis of Metabolic Syndrome

We used the criteria from the NCEP-ATP III [2] for the diagnosis of MetS for women: Waist circumference ≥88 cm (abdominal obesity), blood pressure elevation (≥130/85 mmHg), low HDL-c (<50 mg/dL), high triglycerides (≥150 mg/dL), and hyperglycemia (fasting glucose ≥110 mg/dL or a previous diagnosis of diabetes). Metabolic syndrome was diagnosed when women had three or more of the above mentioned criteria.

### 4.3. Biochemical Assays

Serum levels of glucose, total cholesterol, triglycerides, HDL-c and LDL-c were determined using conventional enzymatic colorimetric methods with commercially available kits (Spinreact, S.A. Girona, Spain), following the instructions of the manufacturers.

### 4.4. Genomic DNA Isolation and Genotyping

Genomic DNA was extracted from peripheral blood leukocytes using the nonenzymatic rapid technique [30]. Six SNPs were genotyped in the *ESR1* gene (rs1884051, rs2077647, rs3798577, rs1801132, rs2234693, rs9340799), two in the *LPL* gene (rs328 and rs320) and one in the *CETP* gene (rs708272), using the 5′-nuclease assay for real time polymerase chain reaction (RT-PCR). Briefly, a reaction mixture was prepared with 10 µL of master mix and 0.5 µL TaqMan probe 20X specific to each SNP (Life Technology, Applied Biosystems, Grand Island, NY, USA), of this reaction mixture, 15 µL were placed in each well of the plate, adding 5 µL of DNA previously extracted of each sample, for a total volume of 20 µL. The reaction was performed in automated equipment 7500 RT-PCR System (Applied Biosystems, USA). The amplification program consists of a Hold at 95 °C for 10 min, 40 cycles at 92 °C for 15 s and 60 °C for 1 min. In 10% of randomly selected samples, genotyping was performed in duplicate and 100% concordance was found.

### 4.5. Ancestry Informative Markers

In order to reduce population stratification bias, a panel of ancestry informative markers (AIMs) was determined. The AIMs used in this study were biallelic SNPs that were selected with Affymetrix Human 100K chip, based on the available information for ancestry in the ancestral population samples genotyped. Because our population is a mixture of the three ancestral populations: African, European and Native American, SNPs were selected if the difference in allele frequency (delta value) was at least 0.5 (scale of 0–1) between either ancestral populations. One-hundred and four AIMs were selected, which were distributed across the genome, a physical distance between them such that were in linkage equilibrium in the three ancestral populations (distance between markers was about 2.4 × 107 bp). Genotyping was performed using iPLEX reagents and protocols for multiplex PCR, single base primer extension (SBE) and generation of mass spectra, as per the manufacturer’s instructions (Sequenom, San Diego, CA, USA). Multiplexed PCR was performed in 5-µL reactions on 384-well plates containing 5 ng of genomic DNA. Reactions contained 0.5 U HotStarTaq polymerase (QIAGEN Company, Venlo, The Netherlands), 100 nM primers, 1.25× HotStar Taq buffer, 1.625 mM MgCl_2_, and 500 µM dNTPs. Following enzyme activation at 94 °C for 15 min, DNA was amplified with 45 cycles of 94 °C × 20 s, 56 °C × 30 s, 72 °C × 1 min, followed by a 3-min extension at 72 °C. Unincorporated dNTPs were removed using shrimp alkaline phosphatase (0.3 U, Sequenom). Single-base extension was carried out by addition of SBE primers at concentrations from 0.625 µM (low MW primers) to 1.25 µM (high MW primers) using iPLEX enzyme and buffers (Sequenom) in 9-µL reactions. Reactions were desalted and SBE products measured using the MassARRAY Compact system, and mass spectra analyzed using TYPER software (Sequenom), in order to generate genotype calls and allele frequencies.

### 4.6. Statistical Analysis

Data are presented as median (25th–75th percentile), or percentage of total for qualitative variables. To compare medians and frequencies between groups, Mann Whitney or Chi-square (*X*^2^) tests were used. Hardy-Weinberg equilibrium (HWE) was verified using the *X*^2^ test with one degree of freedom. Haplotype frequencies and linkage disequilibrium was calculated using the genetic data analysis program SNPstats (http://bioinfo.iconcologia.net/SNPstats). Logistic regression models were adjusted for age, years of schooling and by the admixture proportion (Native American, European and African) to assess the association between the different SNPs, haplotypes and AIMs in women with or without MetS. Statistical analysis was performed using STATA software 11.2 (StataCorp, Lakeway Dr, College Station, TX, USA). Significance level uncorrected (*p* < 0.05) or corrected (*p* < 0.01) for the analysis of SNPs based on the method of correction for multiple comparisons were used, method proposed by Cheverud [31].

## 5. Conclusions

In conclusion, our results show the participation of the variants in the *ESR1*, *LPL* and *CETP* genes in metabolic events related to MetS or some of its features in a Mexican mestizo population. These results suggest that SNPs in these genes may be involved in mechanisms related to MetS, such as lipid and carbohydrate metabolism, and could contribute to metabolic abnormalities.
